# Editorial: New Training Strategies and Evaluation Methods for Improving Health and Physical Performance

**DOI:** 10.3390/ijerph19105855

**Published:** 2022-05-11

**Authors:** Catarina N. Matias, Stefania Toselli, Cristina P. Monteiro, Francesco Campa

**Affiliations:** 1 Bettery Life Lab, Innovation Direction, Bettery S.A., 2740-262 Lisboa, Portugal; katarina_matias@hotmail.com; 2 Centro de Investigação em Desporto, Educação Física, Exercício e Saúde, Universidade Lusófona, 1749-024 Lisboa, Portugal; 3 Department of Biomedical and Neuromotor Sciences, University of Bologna, 40126 Bologna, Italy; stefania.toselli@unibo.it; 4 Interdisciplinary Center for the Study of Human Performance (CIPER), Faculdade de Motricidade Humana, Universidade de Lisboa, 1495-761 Cruz-Quebrada, Portugal; cmonteiro@fmh.ulisboa.pt; 5 Laboratory of Physiology and Biochemistry of Exercise, Faculdade de Motricidade Humana, Universidade de Lisboa, 1495-761 Cruz-Quebrada, Portugal; 6 Department for Life Quality Studies, University of Bologna, 47921 Rimini, Italy; 7 Department of Biomedical Sciences, University of Padua, 35131 Padua, Italy

## 1. Introduction

Physical activity is among the most effective methods for improving health, body composition, and physical function, and its practice is suitable for every population [1,2]. Its benefits are known for sedentary individuals who, by initiating sport, improve their physical condition by reducing risk factors [2]. Active training is also encouraged for the general population who need to maintain an optimal level of fitness, as well as for athletes who want to achieve high performance during competitive periods. Even young people benefit from sports practice, growing into healthy young adults with important implications for their psychological and social development [1,2]. 

In the last few years, the scope of research in sports has become very wide and detailed, laying the foundations for the development of innovative training methods and new evaluation approaches aimed at improving health, body composition, and performance [3,4,5]. Contemporary researchers have contributed to the field of body composition research in the development of new measurement methods and training strategies [6,7]. The aforementioned aspects have laid the foundations for the development of innovative techniques and new evaluation approaches aimed at improving and assessing body composition and sports performance. In these contexts, the bioelectrical impedance analysis was proposed as a valid method to quantify body composition elements (e.g., fat and fat-free mass, body fluids, muscle mass) and are based on predictive equations or the qualitative interpretation of the raw data [8,9,10,11]. On the other hand, innovative training strategies aimed at improving body composition and performance have been presented [5,12]. 

The aim of this Special Issue was to propose, on the basis of the evidence that the current literature provides, new training techniques and specific evaluation methods for the different populations practicing physical activity. 

## 2. Published Manuscripts 

Most of the articles published in this Special Issue focused on the relation between physical performance and physiological and morphological features [13,14,15,16]. Four papers investigated body composition in sports practice [17,18,19,20], while two articles evaluated new strategies aimed at improving and monitoring the recovery phase after the exercise [21,22]. New findings related to training strategies have been reported in three manuscripts [23,24,25] and evaluation procedures for amputee soccer players have been summarized in a scoping review [26]. 

Antunes et al. [13] reported that faster upper body oxygen uptake and hemoglobin/myoglobin deoxygenation kinetics are not associated with an increased upper body repeated-sprint ability in trained judokas. Villalon-Gasch et al. [14] showed how the use of conditioning activity promotes the vertical jump performance in Post- postactivation performance enhancement (PAPE) tests. Specifically, this ativity has been proposed to be used when high-intensity voluntary conditioning contractions lead to enhancement in voluntary muscular performance, and therefore activation is produced in different ways as with post-activation potentiation. Denby et al. [15] demostrated that wrist percooling during a 10 km time-to-trial in the heat resulted in a faster self-selected running speed and higher heart rates, though thermal sensation or perceptions of effort were unaffected. Valamatos and co-workers [16] identified several biomechanical determinants for sprinters. Particularly, in the “Set” position, an anthropometry-driven block setting facilitating hip extension and a rear leg contribution should be encouraged. At the push-off, a rapid extension of both hips and greater force production seems to be important. Additionally, after block exiting, shorter flight times and greater propulsive forces are the main features of best sprinters.

Regarding body composition and sports practice, Campa et al. [17] clarified the influence of somatotype on bioimpedance vector analysis patterns, showing how mesomorphy was positively associated with the bioelecrical phase angle, while an inverse correlation can be found between ectomorphy and phase angle. According to the research study by Paoli and co-workers [18], the circuit-training spot reduction, which represents a training protocol aimed to stimulate lipolysis locally, may be an efficient strategy to reduce in a localized manner abdominal subcutaneous fat tissue depot. With a randomized controlled trial, Pardo et al. [19] highlighted that the effect of a gerontogymnastics program on cardiometabolic risk factors is mediated by sarcopenia in overweight and obese older women. Lastly, Gobbo et al. [20] discussed, for the first time, the ability of the *Specific* bioimpedance vector analysis in tracking body composition adaptations after a training program in military people.

Concerning the recovery phase after exercise, Piras et al. [21] suggested that microcurrent electrical neuromuscular stimulation (MENS) applied before exercise produced an increase in oxygen extraction at muscle microvasculature. In contrast, MENS applied after exercise improved recovery, with the sympathovagal balance shifted toward a state of parasympathetic predominance. Guler et al. [22] observed that balance performance is impaired in soccer players after both aerobic and anaerobic fatigue. According to their results, the impairment of fatigue and balance performance can be considered significant risk factors.

Physical activity and its role in improving health status were considered in four submissions, where new aspects concerning different training strategies have been also highlighted. Kim and co-workers [23] showed that exercise participation had a positive effect on activity restriction, quality of life, and hematopoietic profile in breast cancer survivors. Coratella et al. [24] showed how a wider stance increases thigh muscles’ activity during squat exercises, possibly because of their longer length. In particular, these results suggested how bodybuilders uniquely recruit muscles when performing different squat variations. Normand-Gravier et al. [25] clarified whether researchers when comparing HIIT to other types of programs had utilized equalized protocols.

Finally, the study of Nowak et al. [26] presented a practical and detailed description of the sports performance tests for amputee soccer players, recommending the use of specific tests: the L test and the The Yo-Yo Intermittent Recovery Test Level 1 to assess agility and endurance, respectively.

This research topic emphasizes important findings and recommendations that may be relevant for researchers and coaches in order to identify innovative training strategies and evaluation methods for improving health and sports performance.
